# Utilizing Integrated UHPLC-Q-Exactive Orbitrap-MS, Multivariate Analysis, and Bioactive Evaluation to Distinguish between Wild and Cultivated Niudali (*Millettia speciosa* Champ.)

**DOI:** 10.3390/molecules29040806

**Published:** 2024-02-09

**Authors:** Yuwei Zeng, Qing Yang, Binbin Huang, Ming Chen, Zichang Liang, Zhifeng Zhang, Jianguang Zhang

**Affiliations:** 1 School of Pharmacy, Sichuan College of Traditional Chinese Medicine, Mianyang 621000, China; q1011483485@163.com; 2 Tibetan Plateau Ethnic Medicinal Resources Protection and Utilization Key Laboratory of National Ethnic Affairs Commission of the People’s Republic of China, Southwest Minzu University, Chengdu 610041, China; 3 Qin Zhou Inspection and Testing Center, Qinzhou 535000, China; 4 Qin Zhou Provincial Health School, Qinzhou 535000, China

**Keywords:** *Millettia speciosa*, wild, cultivated, activity evaluation, UHPLC-Q-Exactive Orbitrap-MS

## Abstract

*Millettia speciosa* Champ. (MSCP) enjoys widespread recognition for its culinary and medicinal attributes. Despite the extensive history of MSCP cultivation, the disparities in quality and bioactivity between wild and cultivated varieties have remained unexplored. In this study, 20 wild and cultivated MSCP samples were collected from different regions in China. We embarked on a comprehensive investigation of the chemical constituents found in both wild and cultivated MSCP utilizing UHPLC-Q-Exactive Orbitrap-MS technology and multivariate analysis such as principal component analysis (PCA) and orthogonal partial least squares discriminant analysis (OPLS-DA). In total, 62 chemical components were unequivocally identified or tentatively characterized. Via the multivariate statistical analysis, we successfully pinpointed nine compounds with the potential to serve as chemical markers, enabling the differentiation between wild and cultivated MSCP varieties. Moreover, both genotypes exhibited substantial antioxidant and anti-fatigue properties. The bioactivities of wild MSCP were marginally higher when compared to their cultivated counterparts. This study illuminates the impressive antioxidant and anti-fatigue potential present in both wild and cultivated MSCP genotypes, further augmenting the allure of this species and opening new avenues for the economic valorization of MSCP. Hence, this study provides a valuable method for the identification and quality control of MSCP and a method in chemistry and pharmacology to assess an alternative possibility for cultivated MSCP.

## 1. Introduction

*Millettia speciosa* Champ. (MSCP), known as Niudali in southern China, derives its name from the belief that it imparts increased strength or power upon consumption. Its earliest recorded mention can be traced back to the “Records of Medicinal Plants in Lingnan”. MSCP is primarily found in southern China, including regions such as Guangdong, Guangxi, Hainan, and Taiwan Province, and it belongs to the Leguminosae family [1]. Its primary medicinal efficacy lies in kidney tonification and essence strengthening, making it a valuable resource for recovery from weakened conditions after illness [2]. Additionally, the MSCP root is commonly used in southern Chinese cuisine and traditional medicine as a soup and wine ingredient, known for its immune-boosting, strength-recovering, and anti-fatigue properties [3].

Phytochemical investigations have unveiled a rich array of bioactive constituents within MSCP, including terpenoids, flavonoids, alkaloids, and phenylpropanoids [1,4,5]. As awareness of the effectiveness of MSCP has grown, it has found diverse applications in various products, including dietary supplements, wines, teas, medicines, health products, and beverages [6,7]. However, the overharvesting of wild MSCP has led to a significant decline in its natural resources, failing to meet the increasing market demand. Consequently, the cultivation of MSCP has gained prominence in China, with cultivated MSCP becoming a major market source.

**Figure 1:**
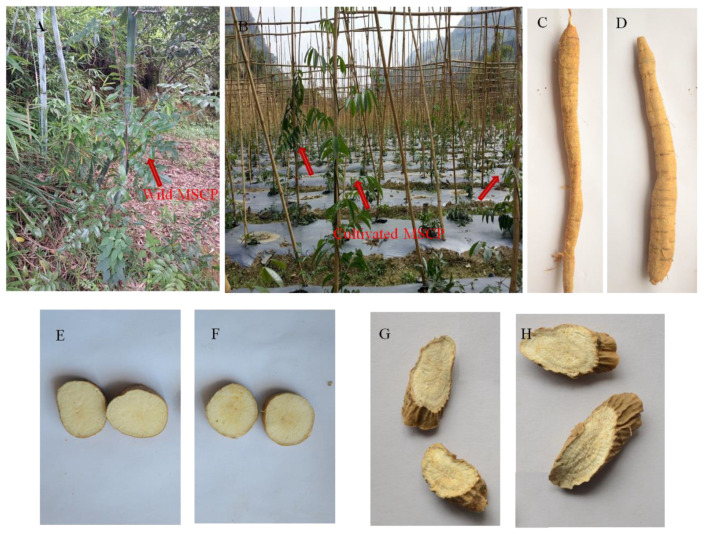
The growing environment of wild MSCP (**A**) and cultivated MSCP (**B**); the overall characteristics of root from wild MSCP (**C**) and cultivated MSCP (**D**); the characteristics of fresh transection from wild MSCP (**E**) and cultivated MSCP (**F**); the characteristics of dried transection from wild MSCP (**G**) and cultivated MSCP (**H**).

Figure 1A illustrates that wild MSCP typically thrives in hillside locations, sparse forests, shrublands, and deep mountain valleys. Wild MSCP stems tend to climb, with the main stem extending 2 to 5 m due to their affinity for sunlight. In contrast, as shown in Figure 1B, cultivated MSCP often grows vigorously sideways due to artificial cultivation practices. Distinguishing between the roots of wild and cultivated MSCP (Figure 1C–H) can be challenging. This distinction is essential because the ecological environments of cultivated and wild MSCP differ significantly, leading to variations in metabolites and their contents. These differences can result in uneven quality and affect pesticide efficacy. Previous research using HPLC-UV revealed higher contents of the three main bioactive compounds in wild MSCP compared to cultivated MSCP [8]. However, relying solely on a few components is insufficient for a comprehensive quality evaluation of herbal medicine, especially for multicomponent and multitarget medicinal herbs. An attempt was made to assess the differences between wild and cultivated MSCP using HPLC fingerprinting [9], but UV detectors have limitations in detecting compounds without UV absorption. Hence, there is a pressing need to establish a comprehensive evaluation method for investigating the distinctions between wild and cultivated MSCP.

Ultra-high-performance liquid chromatography coupled with quadrupole-Orbitrap mass spectrometry (UHPLC-Q-Exactive Orbitrap-MS) has the advantages of excellent sensitivity, wide analytical range, better separation, and reliable qualitative analysis without elaborate sample preparatory procedures, which has been extensively used in identification of herbal medicines [10,11]. It can also be widely applied to identify components from different base resources and parts of traditional Chinese medicine [12,13,14,15]. Moreover, it can also further improve the resolution of unknown compounds, especially for poor chromatographic separation. The antioxidant capacity of foods plays an important role in the diet, which is related to anticancer and anti-aging effects, improvement in immune function, and the prevention of cardiovascular diseases and nervous system diseases [16]. Previous studies have indicated that MSCP had strong antioxidant activity [17] and anti-fatigue activity [18]. However, there are no reports about the differences in the chemical constituents and activity evaluation between wild and cultivated MSCP.

In this work, UHPLC-Q-Exactive Orbitrap-MS was used for rapidly identifying chemical constituents of wild and cultivated MSCP. Multivariate statistical analysis, including principal component analysis (PCA) and orthogonal partial least squares discriminant analysis (OPLS-DA), was used to analyze differences and discover potential characteristic markers between wild and cultivated MSCP. In addition, their potential activity was assessed via antioxidant and anti-fatigue assays.

## 2. Results and Discussion

### 2.1. Identification of Compounds

**Figure 2:**
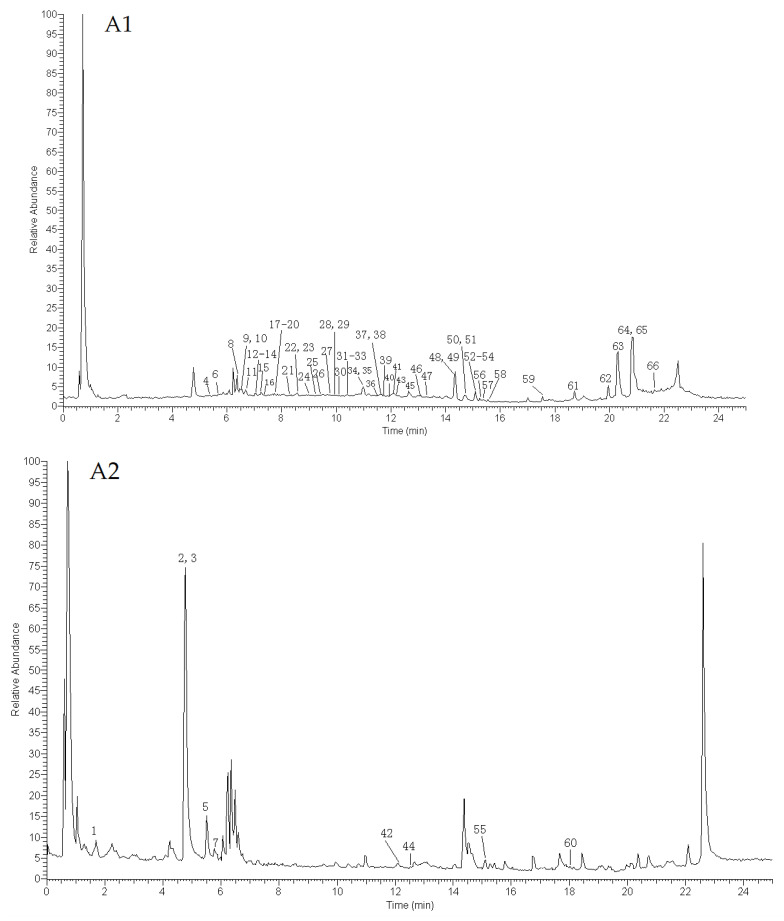

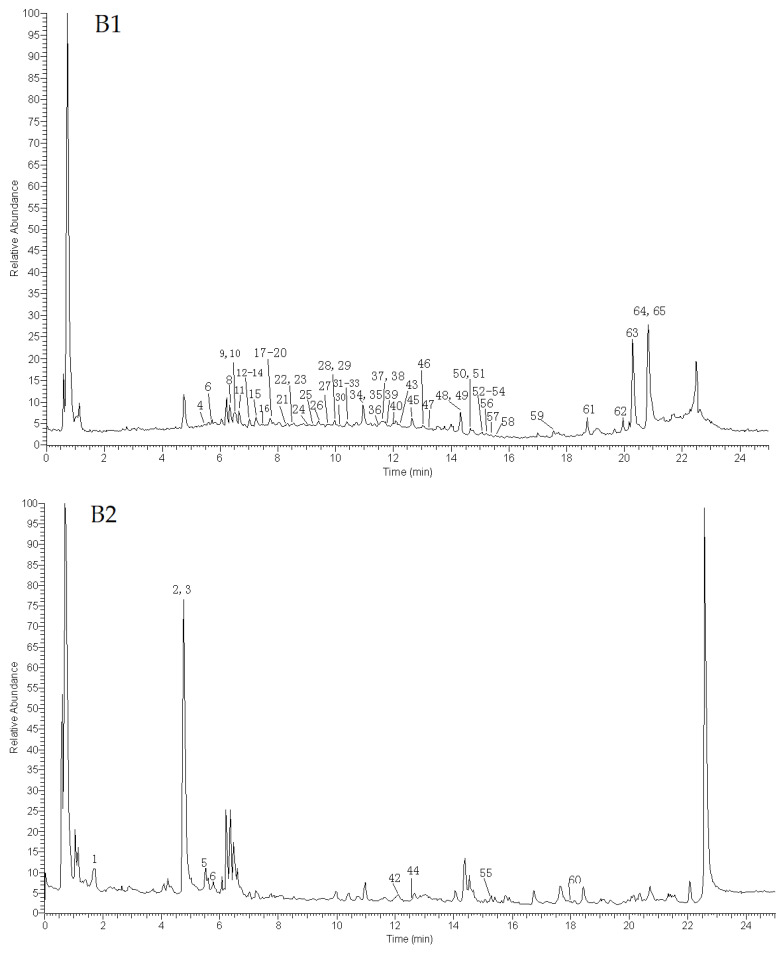
Total ion chromatogram (TIC) profile of wild MSCP ((**A1**) negative ion mode; (**A2**) positive ion mode) and cultivated MSCP ((**B1**) negative ion mode; (**B2**) positive ion mode) based on UHPLC-Q-Exactive-Orbitrap-MS. The peak numbers are the same as in Table 1; peaks without numbers (**A2**,**B2**) are detected and marked in (**A1**,**B1**).

According to the conditions, wild and cultivated MSCP samples were comprehensively analyzed by using UHPLC-Q-Exactive Orbitrap-MS. As shown in Figure 2, alkaloids showed a better signal response in the positive ion mode, but other compounds presented more well in the negative ion mode. There was no significant difference in chemical constituents between the wild and cultivated MSCP samples, and a total of 66 peaks were detected. As listed in Table 1, a total of 62 compounds were tentatively or unambiguously identified according to precise molecular weight, fragmentation ions, fragmentation pathways, and the reported relevant literature [4,5,19,20,21,22,23,24,25,26,27,28], including 36 terpenoids, 16 flavonoids, 4 alkaloids, 2 rotenoids, and 4 others. Among them, five compounds were unambiguously confirmed by comparing them with the reference substance.

#### 2.1.1. Identification of Terpenoids 

**Figure 3:**
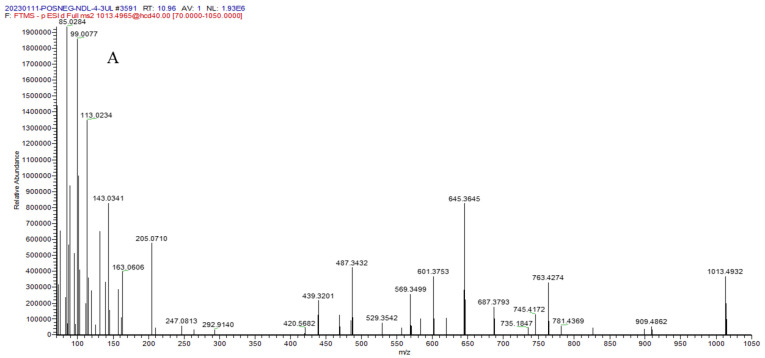

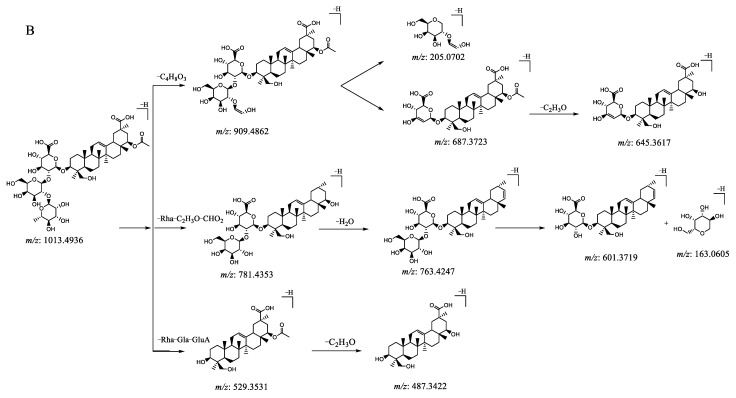
The MS/MS spectrum (**A**) and fragmentation pathway (**B**) of compound **34**.

A lot of terpenoid saponins are isolated from Leguminosae species. In this study, 36 terpenoids were tentatively identified according to MS data, fragmentation ions, and the reported literature. Saponins usually lose one or several sugar moieties (deoxyhexose, hexose, or pentose; *m/z* 132, 146, or 162), carboxyl group, and water group and generate fragmentation ions in the negative mode. Taking compound **34** for the explanation in detail, compound **34** yielded [M − H]^−^ ion at *m/z* 1013.4932, and its chemical formula was speculated to be C_50_H_78_O_21_. It further produced fragment ions at *m/z* 909.4862 [M − H-104]^−^, 781.4369 [M − H-146-43-45]^−^, 763.4274 [M − H-146-43-45-18]^−^, and 601.3753 [M − H-146-43-45-18-162]^−^, and the fragment ion *m/z* 529.3542 belonged to the aglycone ion of terpenoid aglycones, indicating that compound **34** had one deoxyhexose, one hexose, and one glucuronic acid moiety. Compound **34** was tentatively named millettiasaponin A, which was isolated from MSCP according to the reported literature [19]. The mass spectrum and possible cleavage pathway of compound **34** are presented in Figure 3. Compound **23** had [M − H]^−^ ion at *m/z* 793.4055, and its chemical formula was speculated to be C_42_H_66_O_14_. The main fragment ions at *m/z* 599.3633 [M − H-176-18]^−^, 437.3063 [M − H-176-18-162]^−^ were detected, and it was tentatively predicted as calenduloside F. In a similar manner, another seventeen terpenoid saponin compounds (**8**, **9**, **10**, **11**, **19**, **20**, **27**, **29**, **32**, **38**, **41**, **49**, **51**, **53**, **54**, **56,** and **57**) were tentatively confirmed according to the mass spectrometry, fragmentation ions, and the reported literature [5,19].

Many isomers have been identified in MSCP because of the many types of triterpenoid aglycones [5]. For example, compound **14** and compound **15** displayed [M − H]^−^ ions at *m/z* 1103.5643 and 1103.5631, respectively, and they were supposed to be isomers with the same molecular formula C_54_H_88_O_23_. Compound **14** further showed the characteristic fragment ions at *m/z* 895.5084 [M − H-162-46]^−^, 779.4471 [M − H-162-162]^−^, and 571.3987 [M − H-162-46-162- 162]^−^ corresponding to the loss of one glucosyl and HCOOH, two glucosyl, and two glucosyl and HCOOH, respectively. The characteristic fragment ion *m/z* 455.3559 [M − H]^−^ was terpenoid aglycone, and compound **14** was tentatively named oleanolic acid 3-O-β-D-glucopyranosyl-(1→6)-β-d-glucopyranosyl-(1→6)-β-d-glucopyranosyl-28-O-β-D-glucopyranoside [5]. Compound **15** further yielded fragment ions *m/z* 957.5154 [M − H-146]^−^, 777.4453 [M − H-146-162-18]^−^, 616.3993 [M − H-146-162-162-17]^−^, 457.3670 [M − H-146-162-176-162]^−^, and it was tentatively speculated to be soyasapogrnol B 3-*O*-α-l-rhamnopyranosyl-(1→2)-β-d-galactopyranosyl-(1→2)-glucuronopyranosyl-22-*O*-β-d-glucopyranoside [5]. In a similar way, other isomers (compounds **21** and **25**; compounds **13**, **31,** and **47**; compounds **39** and **43**; compounds **48** and **50**; compounds **62** and **63**; compounds **64**, **65,** and **66**) were tentatively identified [5,20]. Compound **61** displayed [M − H]^−^ ion at *m/z* 455.3533, and its chemical formula was speculated to be C_30_H_48_O_3_. Compound **61** was tentatively speculated to be betulinic acid.

#### 2.1.2. Identification of Flavonoids

The retro-Diels–Alder (RDA) reaction and cleavage of glycoside are the main fragmentation patterns of flavonoids [29]. In this work, 16 flavonoids were tentatively identified, including night flavonols, two dihydroflavonoids, three chalcones, and two pterocarpans.

##### Identification of Flavonols

**Figure 4:**
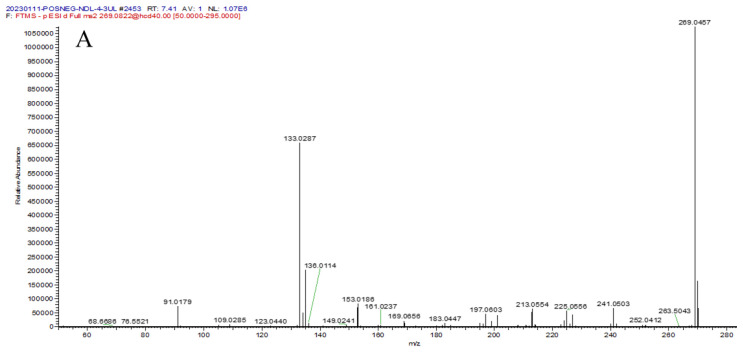

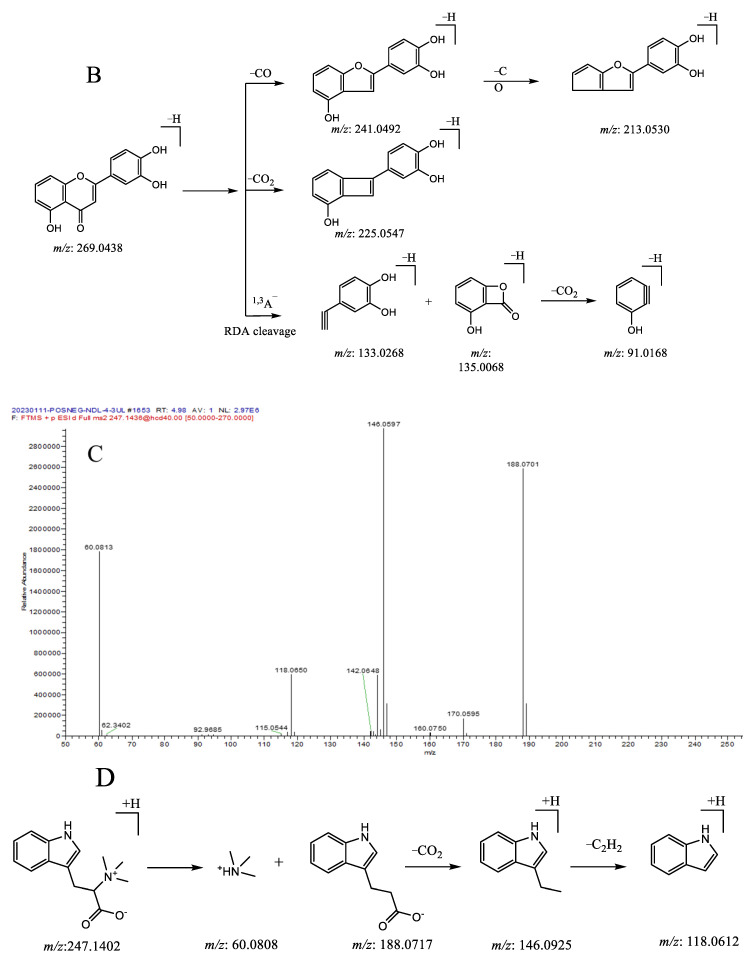
The MS/MS spectrum (**A**) and fragmentation pathway (**B**) of compound **16**; the MS/MS spectrum (**C**) and fragmentation pathway (**D**) of compound **3**.

Compounds **12** and **16** yielded [M − H]^−^ ions at *m/z* 269.0458 and 269.0457 with the same formula: C_15_H_10_O_5_. They produced characteristic fragment ions at *m/z* 241.0505 and 225.0556 by the loss of CO (28 Da) and CO_2_ (44 Da), respectively. Compound **12** further generated a fragment ion at *m/z* 197.0605, and it was tentatively predicted to be baicalein according to the reported literature [21]. However, compound **16** further presented fragment ions at *m/z* 135.0073 (^1,3^A^−^) and 133.02187(^1,3^B^−^) by the RDA cleavage, meaning that ring-A contained one hydroxy group and ring-B had two hydroxy groups. Therefore, compound **16** was tentatively deduced as 5,3′,4′-trihydroxy-flavone [5]. The mass spectrometry and fragmentation process of compound **16** are presented in Figure 4A,B. Compounds **17** and **30** exhibited [M − H]^−^ ions at *m*/*z* 283.0615 and 283.0613, respectively, and their chemical formula was predicted to be C_16_H_12_O_5_. They further produced fragment ions at *m/z* 268.0380 and 240.0424 by successive loss of (15 Da) and CO (28 Da) neutral group in MS^2^ spectra. The characteristic fragment ion at *m*/*z* 211.0396 [M − H-CO_2_-CO]^−^ was detected in compound **17**, while the characteristic fragment ions *m*/*z* 135.0073 (^1,3^A^−^) and 148.0157 (^1,3^B^−^) generated from RDA cleavage were detected in compound **30**. Thus, compounds **17** and **30** were tentatively named isoprunetin [4] and calycosin [22], respectively. Compounds **28** and **33** had [M − H]^−^ ions at *m/z* 299.0564 and 299.0565, respectively. Compound **28** further generated the characteristic fragment ions *m*/*z* 137.0236 (^0,4^A^−^) and 161.0240 (^0,4^B^−^) by RDA cleavage, while compound **33** yielded the characteristic fragment ions at *m*/*z* 151.0070 (^1,3^A^−^) and 148.0156 (^1,3^B^−^). Therefore, compounds **28** and **33** were tentatively speculated to be tectorigenin [4] and pratensein [4], respectively. Compound **36** had [M − H]^−^ ion at m/z 281.0457, whose chemical formula was predicted to be C_16_H_10_O_5_. It further produced the characteristic fragment ions at 135.0089 (^1,3^A-CH_3_) and 117.0339 (^1,3^B- CH_3_) generated from RDA cleavage. Therefore, compound **36** was tentatively speculated to be 7,4′-dimethoxyisoflavone [5]. Compound **40** had [M − H]^−^ ion at *m/z* 267.0665, and the fragment ions *m*/*z* 252.0429, 223.0397, 135.0080, and 132.0287 were further detected. Compound **40** was definitely confirmed as formononetin by comparison with the reference substance [23]. Compound **42** exhibited [M + H]^+^ ion at *m/z* 299.0905, and its chemical formula was speculated to be C_17_H_14_O_5_. It further produced fragment ions at *m/z* 284.0677 and 256.0720 by successive loss of CH_3_ (15 Da) and CO (28 Da). In addition, it also produced the characteristic fragment ions at *m*/*z* 167.0335 (^1,3^A^+^) and 132.0568 (^1,3^B^−^) by RDA cleavage, suggesting that ring-A contained one hydroxy group and methoxy group and ring-B had one methoxy group. Compound **42** was tentatively speculated to be alfalone [5].

##### Identification of Chalcones, Dihydroflavonoids, and Pterocarpans

Compound **4** gave [M − H]^−^ ion at *m/z* 581.1526 with the chemical formula C_26_H_30_O_15_, and fragment ions at *m*/*z* 287.0566, 269.0458, 259.0615, 243.0670, 163.0030, 133.0281 and 125.0236 were observed in the MS^2^ spectrum. Compound **4** was tentatively assigned as okanin 4′-α-l-arabinofuranosyl-(1→4)-glucoside. Compound **18** was unambiguously identified as isoliquiritigenin using the reference substance [24]. Compound **22** had [M − H]^−^ ion at *m/z* 269.0820 with the chemical formula C_16_H_14_O_4_, and the fragment ions at *m/z* 254.05722 [M − H − CH_3_]^−^, 225.0553 [M − H − CO_2_]^−^ were detected in the MS^2^ spectrum. So, compound **22** was tentatively assigned as 4,4′-dihydroxy-2′-methoxychalcone [5].

Compounds **24** and **37** were unambiguously identified as naringenin [25] and liquiritigenin [25] by using their reference standards. Compound **26** had [M − H]^−^ ion at *m/z* 269.0822, and its formula was the same as compound **22** (C_16_H_14_O_4_). The characteristic fragment ions at *m*/*z* 133.0287 and 117.0339 were observed, and compound **26** was tentatively speculated to be medicarpin according to the previous literature [5]. Compound **52** yielded [M − H]^−^ ion at *m/z* 283.0616, and it was unambiguously confirmed as maackiain [23] by comparing the reference standard.

#### 2.1.3. Identification of Alkaloids

Compound **2** possessed [M + H]^+^ ion at *m/z* 188.0703, and the chemical formula was assigned to be C_11_H_9_O_2_N. Furthermore, it generated characteristic fragment ions at *m/z* 142.0648 and 118.0650. Compared with the literature, compound **2** was speculated to be 3-indoleacrylic acid. Compound **3** had [M + H]^+^ ion at *m/z* 247.1437, and the chemical formula was speculated to be C_14_H_18_N_2_O_2_. Furthermore, three primary fragment ions at *m*/*z* 188.0701, 146.0594, and 118.0650 were detected in the secondary mass spectrum, with successive loss of N(CH_3_)_3_^+^ (60 Da), CO_2_ (44 Da) and C_2_H_2_ (26 Da). It was unambiguously identified as hypaphorine [26], in agreement with the reference substance. The mass spectrum and hypothetical fragmentation pathway of compound **3** are displayed in Figure 4C,D. Compound **5** gave the [M + H]^+^ ion at *m/z* 201.1385, with the fragment ion at *m/z* 186.1147 in the MS^2^ spectra, and it was tentatively speculated to be dehydrostobadine. Compound **7** gave [M + H]^+^ ion at *m/z* 217.1335, and its chemical formula was assigned to be C_13_H_16_N_2_O. The characteristic fragment ions at *m/z* 202.10956 [M + H − CH_3_]^+^, 186.1142 [M + H-CH_3_-O]^+^, and 130.0649 [M + H-CH_3_-C_4_H_9_N]^+^ were observed. Compound **7** was plausibly characterized as adrenoglomerulotropin.

#### 2.1.4. Identification of Rotenoids

Compound **44** possessed [M + H]^+^ ion at *m/z* 299.0906, and its chemical formula was the same as compound **42** (C_17_H_14_O_5_), and it was tentatively characterized as millettiaosa A [27]. Compound **58** had [M − H]^−^ ion at *m/z* 283.0955 and [M + Na]^+^ ion at *m/z* 307.0961 with the chemical formula C_17_H_16_O_4_, and it further gave ions at *m/z* 268.0716 [M − H − CH_3_]^−^ in the negative MS^2^ spectrum. Compound **58** was tentatively characterized as millettiaosa B [27].

#### 2.1.5. Identification of Other Compounds

Compound **6** had [M − H]^−^ ion at *m/z* 583.1678, and its chemical formula was speculated to be C_26_H_32_O_15_. Moreover, the fragment ions at *m*/*z* 433.1331, 301.0920, 167.0343, 152.0107, and 123.0443 were detected in the MS^2^ spectrum. Therefore, Compound **6** was tentatively assigned to be seguinoside K [28]. Compound **35** possessed [M − H]^−^ precursor ion at *m/z* 329.2335, suggesting the chemical formula of C_18_H_34_O_5_. Characteristic product ions at *m/z* 229.1443 and 211.1336 were detected, and it was tentatively named 9,12,13-trihydroxyoctadeca-10(E)-dienoic acid [5]. Compound **59** had [M − H]^−^ ion at *m/z* 295.2280, and fragment ions at *m/z* 277.2175 and 171.1018 were observed. Compound **59** was speculated to be 9-hydroxy-10,12-octadecadienoic acid [5]. Compound **60** gave [M + Na]^+^ ion at *m/z* 429.3723, meaning the chemical formula of C_29_H_48_O_2_. Furthermore, it showed characteristic product ions at *m/z* 411.3609, 393.3521, and 369.3146. Therefore, it was tentatively identified as 7-ketositosterol.

### 2.2. Principal Components Analysis (PCA)

**Figure 5:**
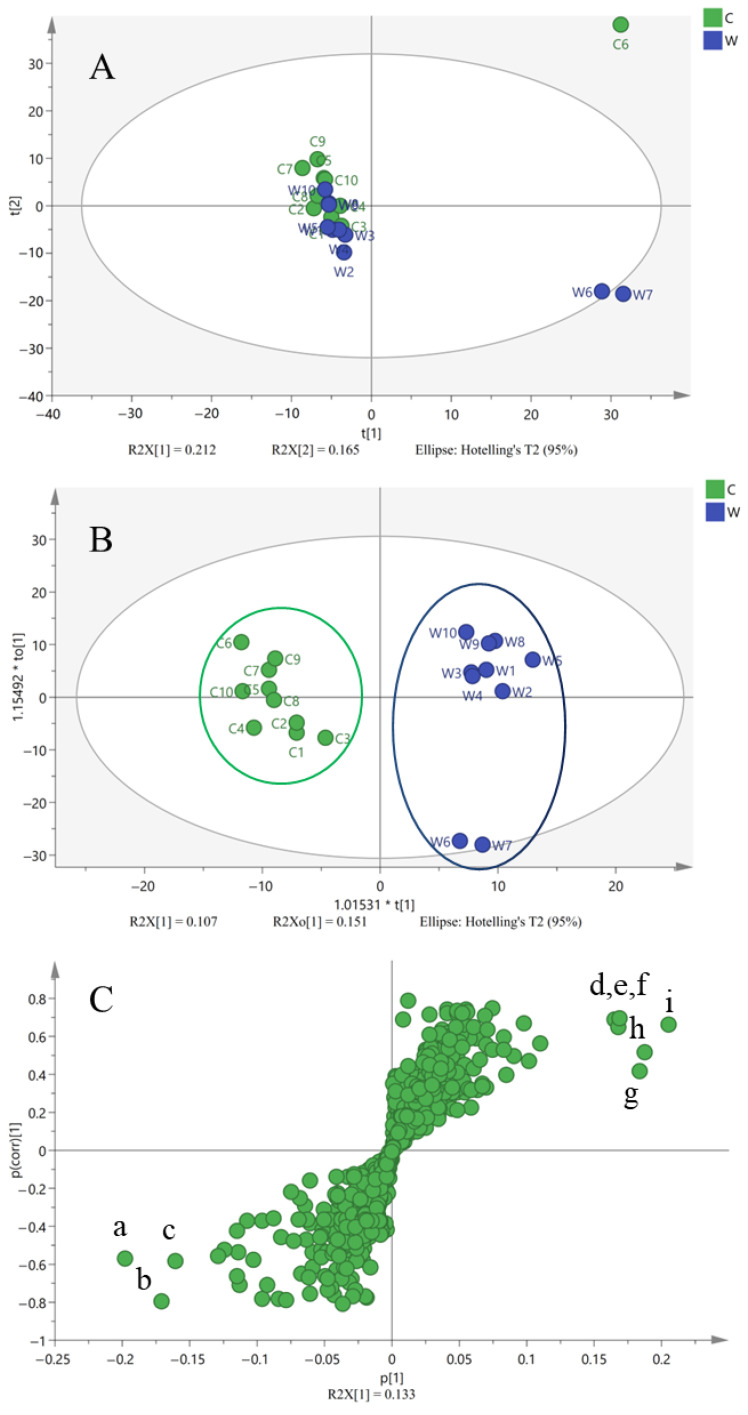
The PCA score plot of wild and cultivated Millettia speciosa samples (**A**); the OPLS-DA score plot showing the discrimination between wild and cultivated Millettia speciosa samples (**B**); and the S-plot score plots (**C**) showing the discrimination of the metabolome of wild and cultivated Millettia speciosa samples (The alphabet a to i stand for potential chemical markers shown in Section 2.3).

From Figure 2, we found that the wild and cultivated MSCP samples had a high degree of similarity in the appearance of TIC and chemical composition. Thus, it is necessary to use multivariable statistics to statistically analyze the MS data and find the differences in components in wild and cultivated MSCP samples. Unsupervised PCA was applied to classify wild and cultivated MSCP samples. The score scatter plot of PCA is shown in Figure 5A. The wild and cultivated MSCP samples were gathered together, indicating that they have great similarities in chemical composition. It also demonstrated that cultivated MSCP was applicable. However, it was difficult to distinguish the wild MSCP from cultivated MSCP by using PCA analysis.

### 2.3. Orthogonal Partial Least Squares Discriminant Analysis (OPLS-DA)

To distinguish and discover chemical markers between wild and cultivated MSCP, OPLS-DA was further applied to analyze the initial MS data. As displayed in Figure 5B, all the wild and cultivated MSCP were divided into two clusters. One cluster, the wild MSCP (W1–W10), is gathered on the right side of the scatter plot, while the other cluster, cultivated MSCP (C1–C10), is gathered on the left side, meaning that there are remarkable differences between wild and cultivated MSCP samples. The S-plot, together with the Variables of Importance in Projection (VIP) value (greater than 2), was then employed to identify the potential chemical markers that presented discrimination between wild and cultivated MSCP samples. A total of nine compounds were identified and marked as potential chemical markers in S-plots (Figure 5C), and the contents of these chemical markers were obviously different between wild and cultivated MSCP samples. Nine compounds generated from S-plots (peak a: 3β-22,24-trihydroxyolean-12-en-29-oic acid 3-*O*-α-l-rhamnopyranosyl-(1→2)-α-l-rhamnopyranosyl-(1→2)-β-d-glucuronopyranoyl-22-*O*-β-d-glucopyranoside, VIP 3.73; peak b: soyasaponin III, VIP 4.94; peak c: soyasaponin VI, VIP 4.48; peak d: 3-*O*-caffeoyloleanolic acid, VIP 4.35; peak e: pyracrenic acid, VIP3.92; peak f: 9-hydroxy-10,12-octadecadienoic acid, VIP 3.90; peak g: betulin-3-caffeate, VIP 3.88; peak h: uvaol-3-caffeate, VIP 3.85; peak i: erythrodiol-3-caffeate, VIP 3.73) were screened to be the potentially characteristic markers for distinguishing the wild from cultivated MSCP samples in accordance with the data from TIC chromatogram. Thus, OPLS-DA combined with VIP values can be used to differentiate the wild from cultivated samples. The OPLS-DA results indicated that there are indeed differences in the contents of these chemical markers between the wild and cultivated MSCP. However, it is necessary to further evaluate whether these differences could induce the change in bioactivity.

### 2.4. Antioxidative Activity Evaluation

Our previous study indicated that MSCP had a strong antioxidant activity [17]. To demonstrate the difference in efficacy of the wild and cultivated MSCP samples, antioxidant activity was evaluated by ABTS and DPPH assays. Both wild and cultivated MSCP samples showed a good antioxidant activity from Table 2. The IC_50_ values of wild and cultivated MSCP were 4.25–6.94 μg/mL and 6.43–10.02 μg/mL in the ABTS assay, respectively. The IC_50_ values of wild and cultivated MSCP were 2.38–5.54 μg/mL and 5.03–8.32 μg/mL in the DPPH assay, respectively. The above results indicated that wild and cultivated MSCP had different degrees of antioxidant capacity. The antioxidant ability is inversely proportional to the IC_50_ value. Therefore, both in ABTS and DPPH assays, the antioxidant activity of the wild MSCP sample was slightly higher than that of the cultivated MSCP samples.

### 2.5. Anti-Fatigue Evaluation

Exercise endurance is a significant variable in evaluating anti-fatigue effects. We constructed an exercise endurance mouse model using a forced swim test to assess the anti-fatigue effect of wild and cultivated MSCP. Moreover, fatigue leads to changes in some biochemical parameters related to fatigue in organisms, including lactic dehydrogenase (LDH), Ca^2+^-Mg^2+^-ATPase (ATPase), superoxide dismutase (SOD) and malondialdehyde (MDA). In this study, LDH activity in the mouse serum and SOD, ATPase, and MDA activity in the mouse liver were measured by kits to assess the anti-fatigue effect of MSCP.

**Figure 6:**
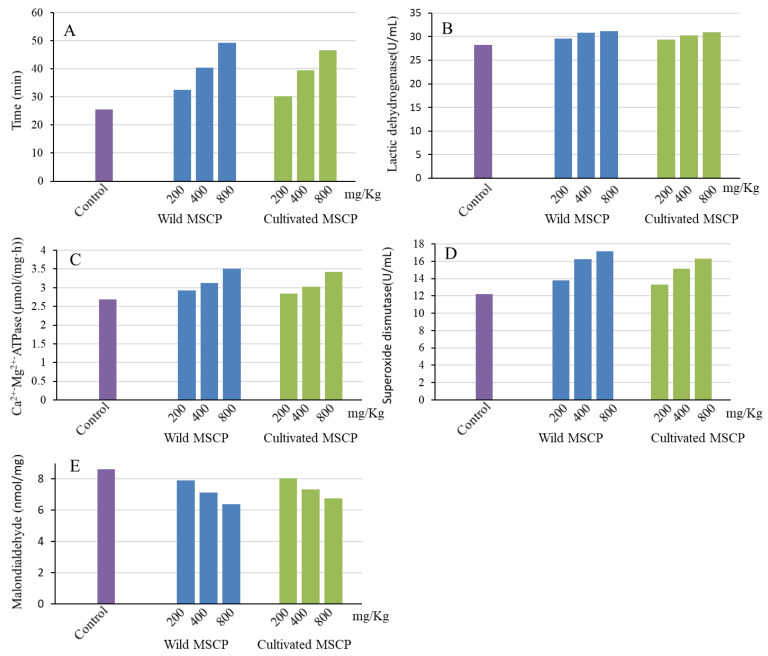
Effect of MSCP on forced swimming time in mice (**A**); the level of lactic dehydrogenase (**B**) in the mouse serum; Ca^2+^-Mg^2+^-ATPase (**C**); superoxide dismutase (**D**); malondialdehyde (**E**) in the mouse liver. control group (physiologic saline, purple column); wild MSCP-treated group (200, 400, and 800 mg/kg body weight, blue column); cultivated MSCP-treated group (200, 400, and 800 mg/kg body weight, light green column).

As shown in Figure 6A, the endurance times in the control, wild MSCP-200, wild MSCP-400, wild MSCP-800, cultivated MSCP-200, cultivated MSCP-400, and cultivated MSCP-800 mg/kg groups were 25.46, 32.52, 40.45, 49.33, 30.18, 39.41, and 46.58 min, respectively. It was indicated that the endurance time of both the wild and cultivated MSCP-800 groups significantly increased (*p* < 0.05) compared with that of the control group. With increasing MSCP content, the endurance time of both wild and cultivated MSCP increased. However, the endurance times of all three wild MSCP groups exhibited slightly longer than those of the same cultivated MSCP content.

As shown in Figure 6B, after swimming, the LDH contents of both the wild and cultivated MSCP groups were higher than that of the control group, and the LDH activity of both wild and cultivated MSCP increased with the increase in the MSCP content. It was also found that the LDH activity of the MSCP-800 group (wild 31.12 ± 3.22 U/mL, cultivated 30.89 ± 4.39 U/mL) showed a significant increase (*p* < 0.05) when compared with the control group (28.22 ± 3.35 U/mL). However, the difference between the same content of the wild and cultivated MSCP groups was not statistically significant.

As shown in Figure 6C, the ATPase activity was proportional to the MSCP content. Meanwhile, the ATPase activity of the experimental group (wild and cultivated MSCP) was higher than that of the control group. In addition, the ATPase activity of the MSCP-800 group (wild 3.51 ± 0.18 μmol/[mg·h], cultivated 3.42 ± 0.21 μmol/[mg·h]) exhibited a significant increase (*p* < 0.05) compared to the control group (2.63 ± 0.43 μmol/[mg·h]). However, all three wild MSCP groups displayed no significant increase (*p* >0.05) compared with the same cultivated MSCP content.

As shown in Figure 6D, the results indicated that the level of SOD increased with increasing MSCP content, and the SOD activity of both the wild and cultivated MSCP groups was higher than that of the control group. In addition, the SOD activity of the MSCP-800 group (wild 17.17 ± 2.18 U/mL, cultivated 16.32 ± 2.41 U/mL) was significantly higher (*p* < 0.05) than that of the control group (12.21 ± 1.31 U/mL). However, there was no significant difference between the wild and cultivated MSCP groups at the same dose.

As shown in Figure 6E, MDA activity was inversely related to the MSCP content. Moreover, the MDA content of both the wild and cultivated MSCP groups was less than that of the control group. The MDA content of the MSCP-800 group (wild 6.39 ± 1.04, cultivated 6.75 ± 0.76 nmol/mg) was obviously decreased (*p* < 0.05) compared to that of the control group (8.62 ± 1.43 nmol/mg). The anti-fatigue effects are inversely correlated with the MDA value; therefore, a lower MDA value showed better exercise tolerance. It was noticed that the MDA activity of cultivated MSCP groups almost showed the same effect compared to wild MSCP with the same content.

These results evidently demonstrated that both wild and cultivated MSCP could enhance the exercise tolerance of mice, and a high dose of both wild and cultivated MSCP showed a significant (*p* < 0.05) anti-fatigue effect on mice with strenuous exercise, but the anti-fatigue effect of wild MSCP was slightly higher than that of the same dose of cultivated MSCP.

### 2.6. The Possibility of Cultivation

According to the results of the above comprehensive study, the possible substitutability of the cultivated MSCP was studied and discussed. The chemical contents in the samples were found to be very similar between the wild and cultivated MSCP. The activity evaluation was also slightly different between wild and cultivated MSCP. Both the antioxidant and anti-fatigue activities of wild MSCP were slightly higher than those of cultivated MSCP. This is powerful evidence for the possible substitutability of cultivated MSCP on the wild MSCP.

## 3. Materials and Methods

### 3.1. Chemicals and Materials

Acetonitrile, methanol, and formic acid were HPLC grade and provided by Merck (Darmstadt, Germany). Reference standards, including hypaphorine, isoliquiritigenin, naringin, formononetin, and maackiain, were of high purity grade (purity > 98%) and purchased from Chengdu Must Biotechnology Co., Ltd. (Chengdu, China). The 2,2′-azino-bis (3-ethylbenzothiazoline-6-sulphonic acid (ABTS) and 1,1-diphenyl-2-picrylhydrazyl (DPPH) were provided by Sigma-Aldrich (St. Louis, MO, USA). Pure water was provided by Wahaha (Hangzhou, China). The kits of lactic dehydrogenase (LDH), Ca^2+^-Mg^2+^-ATPase (ATPase), superoxide dismutase (SOD), and malondialdehyde (MDA) were provided by Nanjing Jiancheng Biological Engineering Research Institute.

### 3.2. Plant Materials

Ten batches of fresh wild MSCP were collected at Qinzhou, Nanning, Shangsi, and Pubei, Guangxi Province. Ten batches of fresh cultivated MSCP for 4 years from the Guangxi and Guangdong Provinces were collected. All plant samples were authenticated by Prof. Hao Zhang (School of Pharmacy, Sichuan University) (Table 3). The fresh sample was cut into smaller pieces and dried in a drying oven at 60 °C and then smashed into powder through 60 meshes for storage in a desiccator.

### 3.3. Preparation of Samples

A total of 0.3 g of sample powder was accurately weighed and put into a 50 mL volumetric flask and then ultrasonically extracted with 10 mL of 70% methanol for 30 min. The ultrasonic power was set at 250 W and the ultrasonic frequency at 40 kHz. After that, the solution was cooled to room temperature, and 70% methanol was added to compensate for the weight lost during extraction. Finally, 0.22 μm of microfiltration membrane was used to filter the extract before UHPLC-Q-Exactive Orbitrap-MS and antioxidant assay.

For the anti-fatigue assay, 200 g of sample powder was extracted with 3000 mL of boiling water for 3 h. Then, the extract was filtered, concentrated, and vacuum-dried at 60 °C to obtain the powder extract. Finally, the powder extract was dissolved in distilled water before oral administration to mice.

### 3.4. Preparation of Standard Solution

For UHPLC-Q-Exactive Orbitrap-MS analysis, five standards, including hypaphorine, isoliquiritigenin, naringin, formononetin, and maackiain, were accurately weighed and dissolved in methanol/water (50% *v*/*v*) to obtain mixed stock solutions with a concentration of 0.1 mg/mL. Then, it was diluted and filtered through 0.22 μm membranes for qualitative analysis.

### 3.5. UHPLC-Q-Exactive Orbitrap-MS Conditions

The UHPLC-Q-Exactive Orbitrap-MS conditions were performed according to our previous study [30]. Chromatographic separation was carried out on a Vanquish Flex UHPLC system (Thermo Fisher Scientific Inc., Waltham, MA, USA) equipped with an ACQUITY HSS T 3 column (100 mm × 2.1 mm, 1.8 μm; Waters, Milford, MA, USA) at 35 °C. The mobile phase consisted of 0.1% aqueous formic acid (A) and acetonitrile (B). The gradient elution conditions were set as follows: 0–1.5 min, 5–8% B; 1.5–3 min, 8–12% B; 3–4.5 min, 12–30% B; 4.5–10 min, 30–35% B; 10–12 min, 35–40% B; 12–15 min, 40–65% B; 15–18 min, 65–85% B; 18–21 min, 85–95% B; 21–21.1 min, 95–5% B and 21.1–25 min, 5–5% B. The flow rate was 0.4 mL/min, and the volume of injection was 3 μL.

Mass data were obtained using a Q-Exactive Orbitrap Mass technology (Thermo Fisher Scientific Inc., USA) equipped with an electrospray ionization (ESI) source. Full-scan MS spectra were monitored in the range of *m*/*z* 100–1500 Da. The capillary voltage was set at 2.8 kV. The source and desolvation temperatures were maintained at 100 and 400 °C, respectively. The cone and desolvation gas flow rates were 20 and 800 L/h, respectively. Xcalibur 2.1 software (Thermo Fisher Scientific Inc., USA) was used to analyze all the mass data.

### 3.6. Antioxidant Activity on ABTS and DPPH

#### 3.6.1. ABTS Radical Scavenging Activity

The ABTS test was conducted as reported by Moon et al. with slight modifications [31]. In brief, 7 mmol/L ABTS aqueous solution and 2.45 mmol/L K_2_S_2_O_8_ were mixed in equal volumes and stored in a dark place for 12 h. The ABTS^+^ analysis solution was obtained from the above ABTS^+^ mixed solution diluted with anhydrous ethanol and read at 734 nm with an absorbance of 0.70 ± 0.02. After that, 0.4 mL of sample solution was mixed with 4 mL of ABTS^+^ solution. After incubation at 25 °C in the dark for 5 min, the absorbance was read at 734 nm using a UV-1800 spectrophotometer (Shimadzu, Kyoto, Japan). The ABTS activity was computed as Equation (1).

ABTS activity (%) = (1 − As/Ab) × 100%(1)

where As is the absorbance of 0.4 mL sample solution, and Ab is using anhydrous ethanol instead of the sample solution. The scavenging activity of the samples was expressed by half inhibition concentration (IC_50_). All tests were determined in triplicate.

#### 3.6.2. DPPH Radical Scavenging Activity

The DPPH scavenging capacity was also determined according to our previous study with a few modifications [32]. An amount of 1 mL of the sample solution was added to 4 mL of 0.04 mg/mL DPPH solution. After 30 min of incubation away from light at 25 °C, the absorbance was recorded at 517 nm using a UV-1800 spectrophotometer (Shimadzu, Japan) to calculate DPPH activity as Equation (2).

DPPH activity (%) = (1 − As/Ab) × 100%(2)

where As is the absorbance of a 1 mL sample solution, and Ab uses anhydrous ethanol instead of the sample solution. The half inhibition concentration (IC_50_) value was used to illustrate the scavenging activity of the samples, and all samples were tested three times.

### 3.7. In Vivo Anti-Fatigue Experiment

#### 3.7.1. Animals and Treatments

Male Kunming mice (6 weeks old, approximately 20 g) were obtained from Chengdu Dashuo Experimental Animal Co., Ltd. (Chengdu, China). All the experiments were performed under the approval of the Animal Ethics Committee of Southwest Minzu University, Chengdu Sichuan, China. Following a 7-day adaptation period, a total of 42 mice were randomly divided into 7 groups, each consisting of 6 mice. Group 1 served as the blank control group and was treated with normal saline. Groups 2–4 were administered low, middle, and high doses of wild MSCP (200, 400, and 800 mg/kg, respectively). Similarly, Groups 5–7 received low, middle, and high doses of cultivated MSCP (200, 400, and 800 mg/kg, respectively). The mice were orally gavaged with 0.2 mL of saline or the corresponding agents for 20 consecutive days. Prior to the forced swim test, the mice underwent adaptive training by swimming for 5 min once a week without any loading. The animal experiments were performed in accordance with the National Research Council’s Guides for the Care and Use of Laboratory Animals.

#### 3.7.2. Forced Swim Test

The forced swim test was evaluated according to the procedure described by Zhao [18] with minor modifications. The forced swim test was performed after forced swim gavage for one hour. The root of each mice’s tail was attached to lead with 5% of body weight, and the mice were put into a swimming tank (90 × 50 × 50 cm, 25 ± 2 °C) with water at a depth of 35 cm. The time from the beginning of the test until the mice failed to return to the water’s surface within 10 s was recorded as the forced swimming time.

#### 3.7.3. Biochemical Assays

After the forced swim test, all the mice were removed from the water. Blood was collected from the orbital venous plexus of mice and centrifuged at 3000 rpm and 4 °C for 10 min, and the supernatant plasma was collected. The LDH activity in serum was determined by using LDH testing kits. Each mouse was euthanized immediately. Liver samples were used to assess ATPase, SOD, and MDA levels by using testing kits.

### 3.8. Data Acquisition and Analysis

The Xcalibur 2.1 software (Thermo Fisher Scientific Inc., Waltham, MA, USA) was employed to process accurate mass data. The extraction parameters were as follows: the match factor was 0.3 ppm; the minimum retention time window and maximum number of peaks were set to 0.05 min and 1,000,000, respectively; the mass tolerance was set to 10 Da; and the noise threshold was set to 100.

SIMCA-P14.1 software (version 14.2, Umetrics, Umeå, Sweden) was extensively applied to preliminarily evaluate the mass data, and it was further used to predict the important components that can distinguish wild from cultivated samples. In this work, unsupervised principal component analysis (PCA) was used to study the variation trend, and orthogonal partial least squares discriminate analysis (OPLS-DA) was used to further differentiate the wild from cultivated MSCP samples. Then, the S-plot combined with the importance in the projection (VIP) was applied to screen out underlying chemical compounds that can distinguish the wild from cultivated MSCP samples.

All experiments were tested three times. All data are expressed as mean ± standard error of the mean and evaluated via one-way ANOVA by using SPSS 22.0 software (SPSS Inc., Chicago, IL, USA).

## 4. Conclusions

In our study, we conducted a comprehensive investigation into the chemical components and biological activities of wild and cultivated Millettia speciosa Champ. (MSCP) for the first time. Subsequently, we compared the antioxidant and anti-fatigue activities of their extracts. Using UPLC-Q-Exactive Orbitrap-MS, a total of 62 compounds were confirmed or tentatively identified. The results of principal component analysis (PCA) indicated that both wild and cultivated MSCP contained similar structural types and chemical components. However, orthogonal partial least squares discriminant analysis (OPLS-DA) revealed discernible differences between wild and cultivated MSCP. We identified nine compounds that could serve as potential chemical markers for distinguishing between wild and cultivated MSCP, including 3β-22,24-trihydroxyolean-12-en-29-oic acid 3-*O*-α-l-rhamnopyranosyl-(1→2)-α-l-rhamnopyranosyl-(1→2)-β-d-glucuronopyranoyl-22-*O*-β-d-glucopyranoside, soyasaponin III, soyasaponin VI, 3-*O*-caffeoyloleanolic acid, pyracrenic acid, 9-hydroxy-10,12-octadecadienoic acid, betulin-3-caffeate, uvaol-3-caffeate, erythrodiol-3-caffeate. Additionally, the antioxidant and anti-fatigue activities of the wild MSCP exhibited slightly stronger than those of the cultivated MSCP. Findings from this work indicate that commercial cultivation of MSCP is promising, as no adverse effects on the efficacy and quality of cultivated MSCP were observed. Moreover, cultivating MSCP could make this product more easily accessible and affordable to consumers while maintaining a similar quality to wild MSCP. Furthermore, it could help avoid the scarcity of wild resources caused by the irrational harvesting of wild plants. These findings provide valuable evidence supporting the potential substitutability of cultivated MSCP for wild varieties and the conservation of wild resources. This study also provides an appropriate method to improve the quality control of MSCP extraction and fundamental chemical and pharmacological effects for potentially replacing wild MSCP.

## Figures and Tables

**Table 1:** The mass spectrometry data and identification results of MSCP by UHPLC-Q-Exactive-Orbitrap-MS.

NO.	Retention Time (min)	ESI-MS (*m*/*z*)	Error (ppm)	MS/MS Fragments Ions	Formula	Identification
1	1.72	254.1609 [M + H]^+^	−0.2	196.1158, 195.1123, 167.1175, 125.0707	C_11_H_19_N_5_O_2_	unknown
2	4.78	188.0703 [M + H]^+^	−1.5	142.0648, 118.0650,	C_11_H_9_O_2_N	3-Indoleacrylic acid
3	4.78	247.1437 [M + H]^+^	−1.4	188.0701, 146.0594, 118.0650, 60.0183	C_14_H_18_O_2_N_2_	Hypaphorine
4	5.45	581.1526 [M − H]^−^	3.4	563.1313, 287.0566, 269.0458, 259.0615, 243.0670, 163.0030, 133.0281, 125.0236	C_26_H_30_O_15_	Okanin 4′-alpha-l-arabinofuranosyl-(1→4)-glucoside
5	5.51	201.1385 [M + H]^+^	−2.8	186.1147	C_13_H_16_N_2_	dehydrostobadine
6	5.67	583.1678 [M − H]^−^	2.6	433.1331, 301.0920, 167.0343, 152.0107, 123.0443	C_26_H_32_O_15_	seguinoside K
7	5.77	217.1335 [M + H]^+^	−0.3	202.10956, 186.1142, 130.0649	C_13_H_16_N_2_O	Adrenoglomerulotropin
8	6.41	1089.5494 [M − H]^−^	−0.2	942.8404, 793.4399, 471.3649	C_53_H_86_O_23_	Soyasapogrnol B 3-*O*-α-l-arabinopyranosyl-(1→2)-β-d-galactopyranosyl-(1→2)-glucuronopyranosyl-22-*O*-β-d-glucopyranoside
9	6.55	1119.5594 [M − H]^−^	1.4	911.4915, 793.4419, 630.0288, 452.1937	C_54_ H_88_O_24_	23-hydroxy-pomalic acid 3-*O*-α-L-rhamnopyranosyl-(1→4)-β-d-glucopyranosyl-(1→6)-β-d-galactopyranosyl-28-*O*-β-d-glucopyranoside
10	6.58	1087.5344 [M − H]^−^	1.8	821.4017, 556.54883, 487.3268, 435.1130	C_53_H_84_O_23_	Oleanolic acid 3-*O*-α-l-rhamnopyranosyl-(1→2)-β-d-glucopyranosyl-(1→2)-β-d-galactopyranosyl-28-*O*-β-d-glucopyranoside
11	6.69	1117.5437 [M − H]^−^	−1.7	1055.5314, 791.4255, 685.3199	C_54_H_86_O_24_	3β,22,24-trihydroxyolean-12-en-29-oic acid 3-*O*-α-l-rhamnopyranosyl-(1→2)-α-l-rhamnopyranosyl-(1→2)-β-d-glucuronopyranoyl-22-*O*-β-d-glucopyranoside
12	6.95	269.0458 [M − H]^−^	4.9	241.0505, 225.0556, 197.0605	C_15_H_10_O_5_	Baicalein
13	7.02	971.4858 [M + HCOO]^−^	1.9	629.3689, 471.3462	C_47_H_74_O_18_	3β-olean-12-en-28,29-dioic acid 3-*O*-α-l-arabinopyranosyl-(1→2)-α-l-rhamnopyranosyl-(1→2)-β-d-galactopyranoside
14	7.02	1103.5643 [M − H]^−^	4.3	895.5084, 777.4471, 571.3987, 455.3559	C_54_H_88_O_23_	Oleanolic acid 3-*O*-β-d-glucopyranosyl- (1→6)-β-d-glucopyranosyl-(1→6)-β-d-glucopyranosyl-28-*O*-β-d-glucopyranoside
15	7.25	1103.5631 [M − H]^−^	−3.2	957.5154, 777.4453, 616.3993, 457.3670	C_54_H_88_O_23_	Soyasapogrnol B 3-*O*-α-l-rhamnopyranosyl-(1→2)-β-d-galactopyranosyl-(1→2)-glucuronopyranosyl-22-*O*-β-d-glucopyranoside
16	7.40	269.0457 [M − H]^−^	4.9	241.0503, 225.0556, 213.0554, 135.0073, 133.0287, 91.0179	C_15_H_10_O_5_	5,3′,4′-trihydroxy-flavone
17	7.73	283.0615 [M − H]^−^	4.4	268.0380, 240.0424, 211.0396	C_16_H_12_O_5_	Isoprunetin
18	7.76	255.0664 [M − H]^−^	4.8	153.0186, 135.0079, 119.0493	C_15_H_12_O_4_	Isoliquiritigenin
19	7.78	1131.5227 [M − H]^−^	−2.2	1090.9832, 805.4061, 536.4517	C_54_H_84_O_25_	3β-olean-12-en-28,29-dioic acid 3-*O*-α-l-rhamnopyranosyl-(1→2)-β-d-galactopyranosyl-(1→2)-glucuronopyranosyl-28-*O*-β-d-glucopyranoside
20	7.81	1073.5536 [M − H]^−^	−2.9	1056.5305, 777.4403, 457.3699	C_52_H_84_O_20_	Oleanolic acid 3-*O*-α-l-arabinopyranosyl-(1→2)-α-l-rhamnopyranosyl-(1→2)-[α-l-arabinopyranosyl-(1→3)]-β-d-galactopyranoside
21	8.26	1071.5377 [M − H]^−^	1.7	1053.5327, 775.4200,435.3287	C_53_H_84_O_22_	Betulinic acid 3-*O*-α-l-arabinopyranosyl-(1→2)-α-l-rhamnopyranosyl-(1→2)-β-d-glucuronopyranosyl-28-*O*-β-d-glucopyranoside
22	8.54	269.0820 [M − H]^−^	4.3	254.05722, 225.0553, 119.0494	C_16_H_14_O_4_	4,4′-dihydroxy-2′-methoxychalcone
23	8.56	793.4055 [M − H]^−^	1.8	599.3633, 437.3063	C_42_H_66_O_14_	Calenduloside F
24	9.08	271.0614 [M − H]^−^	4.7	151.0029, 119.0493	C_15_H_12_O_5_	Naringenin
25	9.32	1071.5386 [M − H]^−^	2.2	775.4217, 435.3235	C_53_H_84_O_22_	Oleanolic acid 3-*O*-α l-arabinopyranosyl-(1→2)-α-l-rhamnopyranosyl-(1→2)-β-d glucuronopyranosyl-28-*O*-β-d-glucopyranoside
26	9.42	269.0822 [M − H]^−^	3.2	254.0581, 175.0394, 161.0237, 133.0287, 117.0339	C_16_H_14_O_4_	Medicarpin
27	9.81	823.4122 [M − H]^−^	1.6	643.3477, 485.3258	C_42_H_64_O_16_	3β-olean-12-en-28,29-dioic acid 3-*O*-β-d-glucopyranosyl-(1→2)-glucuronopyranoside
28	9.92	299.0564 [M − H]^−^	1.2	284.0328, 271.0611, 253.0505, 161.0240, 137.0236	C_16_H_12_O_6_	Tectorigenin
29	9.98	969.4701 [M − H]^−^	−0.9	780.9517, 643.3474, 485.3314, 205.0711, 163.0606	C_48_H_74_O_20_	millettiasaponin B
30	10.09	283.0613 [M − H]^−^	4.7	268.0379, 240.0429, 148.0157, 135.0073	C_16_H_12_O_5_	calycosin
31	10.38	971.4856 [M + HCOO]^−^	−1.4	809.4363, 629.3690, 471.3469	C_47_H_74_O_18_	3α-hydroxy-11-oxoolean-12-en-30-oic acid 3-*O*-α-l-arabinopyranosyl-(1→2)-α-l-rhamnopyranosyl-(1→2)-β-d-galactopyranoside
32	10.42	983.4859 [M − H]^−^	0.8	733.4136, 645.3657, 487.3443	C_49_H_76_O_20_	22β-acetyloxy-3β,24-dihydroxyolean-12-en-29-oic acid 3-*O*-α-l-arabinopyranosyl-(1→2)-α-l-rhamnopyranosyl-(1→2)-[α-l-arabinopyranosyl-(1→3)]-β-d-galactopyranoside
33	10.43	299.0565 [M − H]^−^	1.3	284.0323, 176.0109, 151.0070, 148.0156, 135.0073	C_16_H_12_O_6_	Pratensein
34	10.96	1013.4932 [M − H]^−^	−1.2	909.4862, 781.4369, 763.4274, 687.3793, 645.3645, 601.3753, 529.3542, 487.3432, 205.0710, 163.0606	C_50_H_78_O_21_	millettiasaponin A
35	10.96	329.2335 [M − H]^−^	2.5	229.1443, 211.1336	C_18_H_34_O_5_	9,12,13-trihydroxyoctadeca-10(E)-dienoic acid
36	11.55	281.0457 [M − H]^−^	4.6	251.0350, 225.0555, 135.0079, 117.0339	C_16_H_10_O_5_	7,4′-dimethoxyisoflavone
37	11.63	255.0663 [M − H]^−^	4.8	135.0080, 119.0494	C_15_H_12_O_4_	Liquiritigenin
38	11.66	837.4275 [M + HCOO]^−^	3.6	733.4119, 645.3645, 487.3445, 439.3220	C_42_H_64_O_14_	3α-hydroxy-11-oxoolean-12-en-30-oic acid 3-*O*-α-l-rhamnopyranosyl-(1→2)-β-d-glucuronopyranoside
39	11.74	953.4751 [M − H]^−^	−2.1	627.3541, 537.3569, 469.3323	C_48_H_74_O_19_	3β-olean-12-en-28,29-dioic acid 3-*O*-α-l-arabinopyranosyl-(1→2)-α-l-rhamnopyranosyl-(1→2)-β-d-glucuronopyranoside
40	12.00	267.0665 [M − H]^−^	4.9	252.0429, 223.0397, 135.0080, 132.0287	C_16_ H_12_O_4_	Formononetin
41	12.08	867.4377 [M + HCOO]^−^	3.2	645.3696, 469.3309	C_42_H_62_O_16_	Glycyrrhizic acid
42	12.15	299.0905 [M + H]^+^	4.5	284.0677, 256.0720, 239.0696, 167.0335, 132.0568	C_17_H_14_O_5_	Alfalone
43	12.23	953.4752 [M − H]^−^	2.6	627.3545, 469.3321	C_48_H_74_O_19_	3α-hydroxy-11-oxoolean-12-en-30-oic acid 3-*O*-α-l-rhamnopyranosyl-(1→2)-β-d-galactopyranosyl-(1→2)-glucuronopyranoside
44	12.57	299.0906 [M + H]^+^	4.5	-	C_17_H_14_O_5_	millettiaosa A
45	12.65	997.5020 [M − H]^−^	−0.3	933.3743, 747.4297, 629.3691, 585.3782, 539.3754, 471.3483	C_50_H_78_O_20_	unknown
46	13.06	997.5018 [M − H]^−^	−1.3	747.4376, 629.3703, 585.3818, 539.3750, 471.3483, 443.4824	C_50_H_78_O_20_	unknown
47	13.29	971.4857 [M + HCOO]^−^	−1.4	809.4360, 629.3693, 471.3461	C_47_H_74_O_18_	Saikogenin G 3-*O*-α-l-arabinopyranosyl-(1→2)-α-l-rhamnopyranosyl-(1→2)-β-d-glucuronopyranoside
48	14.32	911.5012 [M − H]^−^	3.5	457.3714, 409.3477	C_47_H_76_O_17_	Soyasaponin II
49	14.33	941.5075 [M − H]^−^	−2.3	457.3685, 426.9266	C_48_H_78_O_18_	Saikogenin G 3-*O*-α-l-rhamnopyranosyl-(1→4)-β-d-glucopyranosyl-(1→6)-β-d-glucopyranoside
50	14.68	911.5014 [M − H]^−^	2.7	472.6508	C_47_H_76_O_17_	Saikogenin G 3-*O*-α-l-arabinopyranosyl- (1→2)-α-l-rhamnopyranosyl-(1→2)-β-d-galactopyranoside
51	14.68	795.4540 [M − H]^−^	−0.2	615.3959, 457.3717	C_42_H_68_O_14_	Soyasapogrnol B 3-*O*-β-d-galactopyranosyl-(1→2)-glucuronopyranoside
52	15.02	283.0615 [M − H]^−^	−0.3	239.0349, 223.0474, 211.0399, 132.0213	C_16_H_12_O_5_	Maackiain
53	15.06	909.4843 [M − H]^−^	−1.2	455.3547, 407.3309	C_47_H_74_ O_17_	3α-hydroxy-11-oxoolean-12-en-30-oic acid 3-*O*-α-l-arabinopyranosyl-(1→2)-β-d-galactopyranosyl-(1→2)-glucuronopyranoside
54	15.09	939.4962 [M − H]^−^	2.1	613.3740, 455.3528	C_47_H_72_O_19_	3α-hydroxy-11-oxoolean-12-en-30-oic acid 3-*O*-α-l-rhamnopyranosyl-(1→4)-β-d-glucopyranosyl-(1→6)-β-d-galactopyranoside
55	15.19	467.1937 [M + Na]^+^	3.4	224.1055, 105.0335	C_27_H_28_N_2_O_4_	unknown
56	15.24	895.5065 [M − H]^−^	4.1	509.4002, 439.3597	C_47_H_76_O_16_	Betulinic acid 3-*O*-α-l-arabinopyranosyl-(1→2)-α-l-rhamnopyranosyl-(1→2)-β-d-galactopyranoside
57	15.32	1067.5437 [M − H]^−^	3.2	921.4910, 583.3988, 457.3726	C_54_H_84_O_21_	Soyasaponin VI
58	15.56	283.0955 [M − H]^−^	−5.5	268.0716, 121.0645	C_17_H_16_O_4_	millettiaosa B
59	17.55	295.2280 [M − H]^−^	1.8	277.2175, 171.1018	C_18_H_32_O_3_	9-hydroxy-10,12-octadecadienoic acid
60	18.02	429.3723 [M + Na]^+^	−1.3	411.3609, 393.3521, 369.3146	C_29_H_48_O_2_	7-Ketositosterol
61	18.77	455.3533 [M − H]^−^	−1.5	-	C_30_H_48_O_3_	Betulinic acid
62	20.07	617.385 [M − H]^−^	2.4	415.2764, 179.0342	C_39_H_54_O_6_	pyracrenic acid
63	20.45	617.3848 [M − H]^−^	2.1	415.2768, 179.0340	C_39_H_54_O_6_	3-*O*-Caffeoyloleanolic acid
64	20.82	603.4056 [M − H]^−^	0.5	179.0338, 161.0237, 133.0284	C_39_H_56_O_5_	erythrodiol-3-caffeate
65	20.92	603.4058 [M − H]^−^	1.0	179.0341, 161.0236, 133.0285	C_39_H_56_O_5_	Betulin-3-caffeate
66	21.67	603.4056 [M − H]^−^	0.7	179.0336, 161.0237, 133.0286	C_39_H_56_O_5_	uvaol-caffeate

**Table 2:** The antioxidant activity (n = 3).

No.	ABTS	DPPH	No.	ABTS	DPPH
	IC_50_ (mg/mL)	IC_50_ (mg/mL)		IC_50_ (mg/mL)	IC_50_ (mg/mL)
W1	6.18 ± 0.21	5.54 ± 0.18	C1	7.18 ± 0.44	6.22 ± 0.21
W2	5.45 ± 0.10	3.23 ± 0.10	C2	7.45 ± 0.18	8.32 ± 0.44
W3	6.94 ± 0.17	4.52 ± 0.22	C3	8.32 ± 0.59	7.25 ± 0.28
W4	5.55 ± 0.31	4.21 ± 0.08	C4	6.62 ± 0.27	5.10 ± 0.17
W5	4.47 ± 0.24	4.33 ± 0.44	C5	8.47 ± 0.34	6.45 ± 0.30
W6	4.69 ± 0.20	3.84 ± 0.12	C6	6.43 ± 0.30	5.03 ± 0.15
W7	5.54 ± 0.31	4.35 ± 0.43	C7	6.74 ± 0.41	7.35 ± 0.36
W8	4.25 ± 0.08	4.26 ± 0.20	C8	7.52 ± 0.16	8.27 ± 0.42
W9	5.57 ± 0.30	5.05 ± 0.24	C9	9.08 ± 0.33	7.17 ± 0.16
W10	6.02 ± 0.31	2.38 ± 0.10	C10	10.02 ± 0.86	6.42 ± 0.25

**Table 3:** Geographical and biological information of 20 MSCP samples.

No.	Origin of Sample	Coordinates	Classification	Collection Date
W1	Longmen, Pubei, Guangxi	N 22°09′53.54″ E 109°22′48.06″	Wild	September 2020
W2	Xiaojiang, Qinzhou, Guangxi	N 22°15′12.86″ E 109°34′25.93″	Wild	September 2020
W3	Naixiao, Nanning, Guangxi	N 22°23′54.99″ E 108°27′43.15″	Wild	September 2020
W4	Yanan, Nanning, Guangxi	N 22°30′7.24″ E 108°08′35.83″	Wild	April 2021
W5	Nanping, Shangsi, Guangxi	N 22°11′27.65″ E 108°03′41.54″	Wild	April 2021
W6	Naqin, Shangsi, Guangxi	N 22°08′6.14″ E 108°04′17.37″	Wild	June 2021
W7	Zhangwang, Pubei, Guangxi	N 22°0′33.95″ E 109°28′47.60″″	Wild	June 2021
W8	Guandong, Pubei, Guangxi	N 22°26′35.47″ E 109°41′19.30″	Wild	October 2021
W9	Duruan, Jiangmen, Guangdong,	N 22°34′30.73″ E 113°02′45.58″	Wild	October 2021
W10	Yayao, Heshan, Guangdong	N 22°42′35.37″ E 113°0′16.14″	Wild	November 2021
C1	Dacheng, Qinzhou, Guangxi	N 22°19′45.46″ E 109°25′20.76″	cultivated	August 2020
C2	Quanshui, Qinzhou, Guangxi	N 21°56′30.57″ E 109°26′53.65″	cultivated	August 2020
C3	Fuwang, Qinzhou, Guangxi	N 22°25′4.50″ E 109°35′18.91″	cultivated	March 2021
C4	Gongzheng, Shangsi, Guangxi	N 22°09′43.45″ E 108°08′35.93″	cultivated	March 2021
C5	Siyang, Shangsi, Guangxi	N 22°07′30.69″ E 108°06′54.38″	cultivated	March 2021
C6	Yanan, Nanning, Guangxi	N 22°22′54.18″ E 108°25′57.85	cultivated	May 2021
C7	Nayang, Hengzhou, Guangxi	N 22°41′57.56″ E 109°19′38.74	cultivated	May 2021
C8	Yayao, Heshan, Guangdong	N 22°42′27.50″ E 112°59′32.86″	cultivated	May 2021
C9	Yayao, Heshan, Guangdong	N 22°42′35.37″ E 113°0′16.14″	cultivated	August 2021
C10	Duruan, Jiangmen, Guangdong	N 22°34′43.13″ E 113°02′41.90″	cultivated	August 2021

## Data Availability

Data are contained within the article.
